# Синдром первичной резистентности к глюкокортикоидам: сложности диагностики (клинический случай и краткий обзор)

**DOI:** 10.14341/probl13321

**Published:** 2024-02-28

**Authors:** И. И. Ларина, Н. В. Маказан, К. В. Иващенко, Н. М. Платонова, Е. М. Орлова, М. А. Карева, Л. С. Созаева, М. Ю. Юкина, А. Н. Тюльпаков, А. С. Духанин, Н. Л. Шимановский, Е. А. Трошина

**Affiliations:** Национальный медицинский исследовательский центр эндокринологии; Национальный медицинский исследовательский центр эндокринологии; Национальный медицинский исследовательский центр эндокринологии; Национальный медицинский исследовательский центр эндокринологии; Национальный медицинский исследовательский центр эндокринологии; Национальный медицинский исследовательский центр эндокринологии; Национальный медицинский исследовательский центр эндокринологии; Национальный медицинский исследовательский центр эндокринологии; Медико-генетический научный центр имени академика Н.П. Бочкова; Российский национальный исследовательский медицинский университет имени Н.И. Пирогова Минздрава России; Российский национальный исследовательский медицинский университет имени Н.И. Пирогова Минздрава России; Национальный медицинский исследовательский центр эндокринологии

**Keywords:** синдром резистентности к глюкокортикоидам, гиперандрогения, клинический случай

## Abstract

Синдром первичной резистентности к глюкокортикоидам (OMIM 615962) — редкое заболевание, характеризующееся генерализованной либо парциальной нечувствительностью органов-мишеней к глюкокортикоидам (ГК). Компенсаторная активация гипоталамо-гипофизарно-надпочечниковой оси приводит к развитию ряда патологических состояний, обусловленных гиперстимуляцией надпочечников. Клинические проявления варьируют от бессимптомного течения до серьезных последствий избытка минералокортикоидов и/или андрогенов. В настоящее время установлена лишь одна причина синдрома резистентности к ГК — инактивирующие мутации в гене рГК NR3C1. Мы представляем клинический случай с периодом наблюдения в 3,5 года, когда клинико-лабораторная картина соответствовала диагнозу синдрома резистентности к глюкокортикоидам, однако мутаций в гене NR3C1 не было обнаружено, а нечувствительность органов-мишеней к ГК была доказана по результатам функционального исследования.

## Abstract (en)

Primary glucocorticoid resistance (OMIM 615962) is a rare endocrinologic condition caused by resistance of the human glucocorticoid receptor (hGR) to glucocorticoids (GR) and characterised by general or partial insensitivity of target organs to GK. Compensatory activation of hypothalamic-pituitary-andrenal axis results in development of a various pathological conditions caused by overstimulation of adrenal glands. Clinical spectrum may range from asymptomatic cases to severe cases of mineralocorticoid and/or androgen excess. At present time, primary generalized glucocorticoid resistance has been exclusively associated with defects in the NR3C1 gene. Here, we present a case report of an adolescent patient with clinical presentation of glucocorticoid resistance confirmed by detailed endocrinologic evaluation but no confirmed mutations in the NR3C1 gene.

## АКТУАЛЬНОСТЬ

Синдром первичной резистентности к глюкокортикоидам (OMIM 615962) характеризуется высоким уровнем кортизола и АКТГ крови в отсутствие характерных кушингоидных черт. При этом могут возникать гипертония, гиперандрогенемия, гипокалиемия, гипогликемия. Каждое проявление синдрома само по себе неспецифично. Когда же резистентность парциальная и клиническая картина стерта, заподозрить и диагностировать это заболевание еще сложнее. При синдроме выявляется гетерозиготная мутация в гене, кодирующем рецептор к ГК (рГК), — NR3C1. Вероятно, это не единственная причина нечувствительности органов-мишеней к ГК. Мы приводим клинический случай, в котором клинико-лабораторные данные соответствовали первичной резистентности к ГК, но мутации в NR3C1 выявлено не было. Для подтверждения нечувствительности к ГК были использованы функциональные методы исследования. Это первое описание подобного случая в России.

## МАТЕРИАЛЫ И МЕТОДЫ

Исследование уровня АКТГ, кортизола, общего тестостерона: иммуноферментный анализ периферической крови.

Исследование уровня ФСГ, ЛГ, эстрадиола, общего тестостерона, АКТГ, АТ к р.ТТГ, ТТГ, свТ3, свТ4, АТ-ТПО, кортизола суточной мочи: хемилюминесцентный иммуноанализ.

Исследование уровня кортизола, АТ к р.ТТГ: электрохемилюминесцентный анализ периферической крови.

Исследование спектра стероидных гормонов и их предшественников: мультистероидный анализ периферической крови.

Молекулярно-генетическое исследование гена NR3C1: полное секвенирование гена по Сэнгеру.

Исследование взаимодействия рГК с кортизолом и дексаметазоном в лимфоцитах периферической крови: радионуклидный анализ в лаборатории нуклеиновых кислот Института молекулярной биологии РАН. Используя меченные тритием кортизол и дексаметазон (3Н-кортизол и 3Н-дексаметазон соответственно), были определены параметры глюкокортикоидной рецепторной системы (аффинность и концентрация рецепторов глюкокортикоидов в клетках), проведено сравнительное исследование взаимодействия природного кортизола и синтетического препарата дексаметазона с рецепторами глюкокортикоидов, изучено комплексообразование ГК с ядрами клеток, определено соотношение между количеством активированной (трансформированной) и неактивной форм цитозольных гормон-рецепторных комплексов (ГРК) на основе измерения доли ядерных рГК от общего пула рецепторов глюкокортикоидов в клетках. Аффинность и концентрация рецепторов глюкокортикоидов в клетках проводилась на цельных лимфоцитах. Для оценки внутриядерного связывания высокомеченных синтетических глюкокортикоидов лимфоциты подвергали гипоосмотическому лизису, затем с помощью дифференциального центрифугирования получали ядра лимфоцитов, в которых измеряли содержание гормон-рецепторных комплексов.

## ОПИСАНИЕ СЛУЧАЯ И РЕЗУЛЬТАТЫ ИССЛЕДОВАНИЙ

Пациентка — девушка 18 лет, наблюдавшаяся в ФГБУ НМИЦ эндокринологии в течение трех лет.

Пациентка была впервые направлена в ФГБУ НМИЦ эндокринологии в возрасте 15 лет с подозрением на болезнь Иценко-Кушинга. Диагноз заподозрили по месту жительства при обследовании по поводу опсоменореи. С момента менархе в 11 лет продолжительность менструального цикла варьировалась от 30 до 90 дней. Также девушку беспокоила угревая сыпь и избыточный рост волос на теле. Угревая сыпь появилась в 5 лет, а темные волосы на руках, ногах и спине — в 11 лет. Было выявлено повышение утреннего кортизола крови до 2152,9 нмоль/л на фоне высоко-нормального уровня АКТГ крови, а также повышение общего тестостерона сыворотки крови (19,4 нмоль/л) и 17-ОН-прогестерона сыворотки крови (22,5 нмоль/л). По результатам МРТ было заподозрено наличие микроаденомы гипофиза. С этими данными девушка была направлена в ФГБУ НМИЦ эндокринологии.

При первичном осмотре в ФГБУ НМИЦ эндокринологии: девушка 15 лет, ростом 163,9 см (SDS роста +0,33), массой тела 61,4 кг (SDS ИМТ +0,93), нормального телосложения. На коже лица, туловища, конечностей — диффузная угревая сыпь (элементы в различной стадии развития), на коже туловища бляшки до 2 см в диаметре, состоящие из мелкопапулезных воспалительных элементов, размещающиеся с образованием гиперпигментированных пятен, 6-й степени по шкале Кука [4]. Рост волос в андроген-зависимых зонах — 6 баллов по шкале Ферримана-Галлвея (избыточный рост волос по белой линии живота (2 балла) периареолярной области (1 балл), бедер (3 балла). Артериальное давление 105/65 мм рт.ст. На момент поступления в отделение задержка менструального цикла более 6 месяцев.

При обследовании подтверждена гиперандрогенемия и гиперкортизолемия. Электролиты крови, активность ренина плазмы и альдостерон — в пределах нормальных значений. По результатам мультистероидного анализа не было получено данных относительно дефектов стероидогенеза или избыток минералокортикоидов. Ночной подавляющий тест с 1 мг дексаметазона привел к снижению уровня кортизола в суточной моче, при этом в крови снижения кортизола достигнуто не было. На фоне большого теста с дексаметазоном секреция кортизола крови снизилась более чем на 60% от изначального. При проведении МРТ головного мозга была выявлена умеренная гиперплазия и диффузная неоднородность структуры аденогипофиза.

Результаты обследования представлены в таблице 1.

**Table:** Таблица 1. Результаты обследования в возрасте 15 лет

Показатель	Результат	Референсные значения
Показатели гормонального профиля
17-ОН-прогестерон, нмоль/л	6,5	0,8–7,0
ДГЭА-С, мкмоль/л	10,00	0,92–7,60
Общий тестостерон, нмоль/л	5,0	0,7–1,8
Эстрадиол, пмоль/л	184,9	97–592
ЛГ, Ед/л	4,2	2,6–12,1
ФСГ, Ед/л	4,3	1,9–11,7
Суточный ритм секреции кортизола и АКТГ
Показатель	Результат
	07:00	23:00
Кортизол, нмоль/л	643,6	46,2
АКТГ, пг/мл	71,5	62,8
Ночной тест с 1 мг дексаметазона
	Базально	На фоне дексаметазона
Кортизол (сыворотка), нмоль/л	643,6	663,8
Малая проба с дексаметазоном (2 дня 2 мг/сут)
	Базально	На фоне дексаметазона
Кортизол (сыворотка), нмоль/л	663,8	719,0
Общий тестостерон, нмоль/л	5,0	3,0
Свободный кортизол в моче, нмоль/сут	2091,0	512,0
Большая проба с дексаметазоном (2 дня 8 мг/сут)
	Базально	На фоне дексаметазона
Кортизол (сыворотка), нмоль/л	719,0	167,4
АКТГ, пг/мл	71,5	44,8
Общий тестостерон, нмоль/л	3,0	1,0
Свободный кортизол в моче, нмоль/сут	567	156

Исследование минеральной плотности костей исключило наличие остеопороза. По данным УЗИ органов малого таза, размеры матки и яичников соответствовали возрастной норме. В связи с отсутствием убедительных данных относительно центрального генеза гиперкортицизма и клинических проявлений синдрома Кушинга была выбрана наблюдательная тактика.

Повторное обследование в 15,7 года не выявило изменений в клинической картине и лабораторных данных. Заподозрен синдром резистентности к глюкокортикоидам и назначено пробное лечение дексаметазоном в начальной дозе 0,5 мг. На фоне терапии дексаметазоном отмечалось снижение уровня андрогенов крови (таблица 2).

**Table:** Таблица 2. Показатели андрогенов крови на фоне назначения терапии дексаметазоном 0,5 мг в сутки

Показатель	До лечения	7 дней терапии дексаметазоном 0,5 мг/сут	Референсный интервал
ДГЭА-С, мкмоль/л	10,39	5,7	0,92–7,60
Общий тестостерон, нмоль/л	5,27	2,5	0,7–1,8

Терапия дексаметазоном продолжалась в течение года, с 15,7 до 16,7 года, суточная доза не превышала 0,75 мг в сутки. Динамика уровней общего тестостерона и ДГЭА-С на фоне терапии дексаметазоном представлена в таблице 3.

**Table:** Таблица 3. Показатели андрогенов крови на фоне терапии дексаметазоном

Длительность терапии Показатели	5 мес	7 мес	10 мес	11 мес	12 мес	1 мес без лечения
Тестостерон, нмоль/л	2,5	18,3	12,3	6,8	5,4	24,2
ДГЭА-С, мкмоль/л	7,70	-	-	-	11,0	-
Свободный кортизол в моче, нмоль/сут	833,0	-	-	-	4400,0	-
Доза дексаметазона, мг/сут	0,5	-	0,75	-	отмена	

При наблюдении в динамике сохранялись нарушения менструального цикла. На фоне избыточной массы тела (SDS ИМТ +1,4) овал лица округлился, что было расценено как стертое проявление синдрома Кушинга. Дексаметазон был отменен в 16,7 года.

С 16 лет, одновременно с приемом дексаметазона, был назначен нестероидный антагонист андрогеновых рецепторов — флутамид. После инициации терапии флутамидом проявления угревой сыпи значительно уменьшились. С 16,9 года менструальный цикл стал регулярным. В таблице 4 приведены сводные данные о клинических проявлениях гиперандрогении с указанием проводимого лечения.

**Table:** Таблица 4. Данные менструального цикла и выраженности угревой сыпи в период наблюдения с 15 до 17,3 года

Период жизни	Терапия (мг в сутки)	Длительность менструального цикла (д.м.ц.) в днях	Интенсивность проявлений угревой сыпи (шкал Кука)
15–15,7 года	-	Вторичная аменорея	6
15,7–16,1 года	Дексаметазон 0,5	Д.м.ц. с 16 лет 27 [ 24; 40]	6
16,1–16,6 года	Дексаметазон 0,75+ флутамид 250	Д.м.ц. 39 [ 29; 50]	2
16,6–16,9 года	Дексаметазон 0,75	Отсутствие менструаций в указанный период	4
16,9–17,3 года	Флутамид 250	Д.м.ц. 33 [ 31; 35]	2

На протяжении всего периода наблюдения у пациентки не отмечалось клинических признаков гиперкортицизма или избытка минералокортикоидов. Вес был в пределах нормы, без патологического распределения подкожно-жировой клетчатки. Сохранялось отсутствие снижения минеральной плотности костей по данным рентгенденситометрии в динамике. Отмечались нормальные показатели АД, электролиты и глюкоза крови были в пределах референсных значений. Тонкие стрии, выявленные при первичном осмотре в 15 лет, располагались в нетипичных для синдрома Кушинга местах и не прогрессировали в динамике.

Помимо функциональных нарушений гипоталамо-гипофизарно-надпочечниковой оси с момента начала наблюдения, отмечалась еще одна особенность — тенденция к снижению тиреотропного гормона (ТТГ). Уровень свободного тироксина (свТ4) варьировал от нормальных до высоких значений, клинических признаков тиреотоксикоза не отмечалось. Значения аутоантител к тиреопероксидазе были в пределах нормы, а уровень антител к рТТГ однократно определялся в «серой зоне» — 1,18 Ед/л. Объем щитовидной железы оставался в пределах нормы. В правой доле отмечалось образование 0,5 см в диаметре без динамики в размерах. Функциональная активность образования была исключена по данным сцинтиграфии щитовидной железы с 99мТс-Технетрилом — индекс захвата технеция составил 0,3%.

В таблице 5 приведены данные о морфофункциональном состоянии щитовидной железы в динамике.

**Table:** Таблица 5. Показатели морфофункционального состояния щитовидной железы в динамике

Данные гормонального профиля и УЗИ	Возраст в годах	
15,5	16	16,3	16,5	16,6	16,7	16,8	16,9	17,3	17,4	23
ТТГ, мМЕ/л	0,3	0,5	0,05	0,12	0,05	0,19	0,105	0,153	0,056	0,116	0,475
свТ4, пмоль/л (референсное значение 10–25)	14,5	12,7	35	29,9	19,8	25,8	3,49	11,1	37,7	12,2	12,17
свТ3, пмоль/л (референсное значение 4–8,5)					9,3		11,14	3,5		3,57	3,48
УЗИ щитовидной железы	В правой доле в нижнем сегменте определяется жидкостная зона д=0,5 см	
Объем щитовидной железы, мл		16,8					18,8			12,8	20,1

Для подтверждения диагноза синдрома резистентности к ГК было проведено молекулярно-генетическое исследование гена NR3C1 и функциональное исследование лимфоцитов периферической крови. Секвенирование гена NR3C1 не выявило патологически значимых изменений нуклеотидной последовательности. Параметры связывания глюкокортикоидов с рГК лимфоцитами периферической крови пациентки существенно не отличались от таковых у контрольных образцов (р <0,05). Результаты радиолигандного анализа связывания 3-Н кортизола лимфоцитами представлены в таблице 6 и на рисунках 1 и 2.

**Table:** Таблица 6. Параметры рецепторного связывания 3Н-кортизола (К) и 3Н-дексаметазона (Д) лимфоцитами периферической крови (М±m)

	Параметры специфического связывания
Кд, нМ	Вмакс, фмоль/10⁶клеток (среднее количество рецепторов на одну клетку)
Резистентность	26,0±1,3 (К) 5,1±0,6 (Д)	13,6±1,7 (К) (9 133) 16,9±1,8 (Д) (8 988)
Контроль	24,2±0,8 (К) 5,7±0,3 (Д)	15,7±1,4 (К) (9 133) 14,9±1,5 (Д) (8 988)

**Figure:**
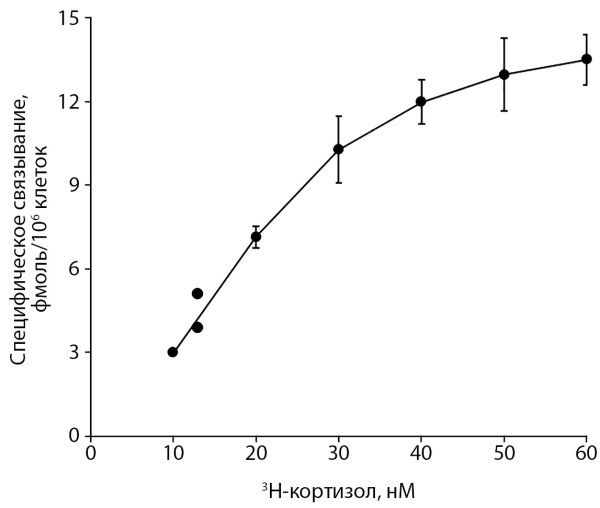
Рисунок 1. Параметры связывания рГК лимфоцитами периферической крови пациентки высокомеченным синтетическим глюкокортикоидом 3Н-кортизолом, представленные в координатах Скэтчарда [5]. Кривая специфического связывания (связывание лиганда с рГК) имеет характерный вид изотермы насыщения, что свидетельствует о наличии в клетках высокоспецифических участков связывания гормона.

**Figure:**
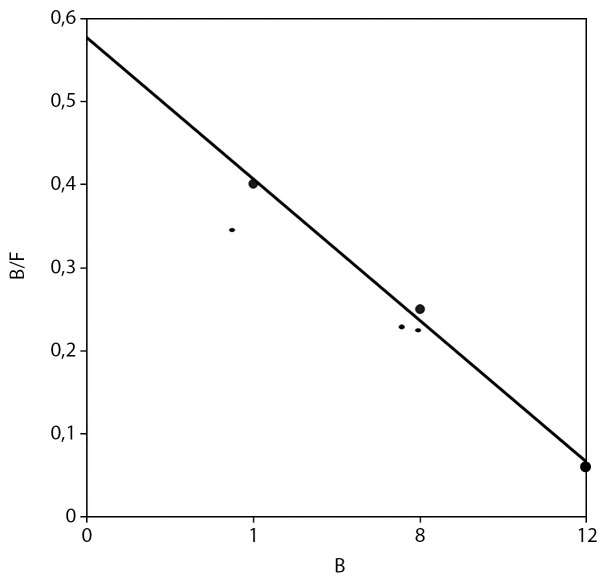
Рисунок 2. Соотношение количества связанного 3H-кортизола (B) и количества свободного (F).

При оценке количества активных гормон-рецепторных комплексов было выявлено сниженное содержание рГК в ядрах лимфоцитов пациентки в сравнении с результатами контрольных образцов. Для 3H-дексаметазона уменьшение составило около 25%, а в случае с 3Н-кортизолом количество ядерных ГР было снижено практически в 2 раза (50%). При этом определение соотношения α-и β изоформ рГК не выявило значимых изменений в их уровне экспрессии. В таблице 7 приведены сведения о содержании цитозольных, ядерных рецепторов глюкокортикоидов в лимфоцитах, а также вычисленное значение соотношения количества ядерных/цитозольных рецепторов.

**Table:** Таблица 7. Соотношение между цитозольными и ядерными рецепторами глюкокортикоидов лимфоцитов периферической крови

	Содержание рецепторов, фмоль/10⁵ клеток	Соотношениеядерные/цитозольные рецепторы
Цитозоль	Ядро	Общее
Резистентность	1,1±0,2 0,6±0,1	0,3±0,1 0,9±0,1	1,4±0,2 (К) 1,7±0,2 (Д)	0,27 1,5
Контроль	0,96±0,2 0,45±0,1	0,6±0,1 1,1±0,1	1,6±0,2 (К) 1,5±0,2 (Д)	0,63 2,44

## ОБСУЖДЕНИЕ

Регуляция гипоталамо-гипофизарно-надпочечниковой оси происходит по механизму отрицательной обратной связи. Снижение кортизола в крови активирует секрецию кортикотропина, тогда как гиперкортизолемия ингибирует синтез кортикотропина [6]. Резистентность к глюкокортикоидам оказывает на функцию гипоталамо-гипофизарно-надпочечниковой оси такое же действие, как низкий уровень кортизола крови — повышается секреция кортикотропина, активируется синтез АКТГ. Это приводит к гиперфункции надпочечников, повышается секреция кортизола, а также — параллельно — андрогенов и минералокортикоидов как побочных продуктов чрезмерно стимулированного стероидогенеза [1][2][7].

Синдром первичной резистентности к глюкокортикоидам был впервые описан в 1976 г. Vingerhoeds A.C. у пациента с гиперкортизолемией и высоким АКТГ. На протяжении трех лет наблюдения отмечались гипокалиемия и артериальная гипертония без характерных проявлений синдрома Кушинга [1]. В 1982 г. Chrousos G.P., Vingerhoeds A.C. et al. провели повторное обследование пациента (пациент 1) и членов его семьи. У сына пациента 1 выявили асимптоматическое повышение кортикостероидов с минералокортикоидной активностью (кортикостерона и дезоксикортикостерона). Резистентность к ГК была доказана на основании сниженного ответа фибробластов на дексаметазон и в дальнейшем подтверждена молекулярно-генетически — детекцией патогенного варианта в гене рецептора к ГК NR3C1 (nuclear receptor subfamily 3, group c, member 1, OMIM 138040) [2][3][19].

У описываемой пациентки отмечалась гиперкортизолемия и высокий уровень АКТГ без характерных для синдрома Кушинга изменений внешности и остеопороза. Не было избытка минералокортикоидов, но подобные варианты течения синдрома резистентности к ГК были описаны ранее [9]. Согласно данным литературы, достичь клинической компенсации заболевания у пациентов с резистентностью к ГК удается при назначении дексаметазона. Эффективность этой терапии у нашей пациентки была сомнительна. Возможно, это было связано с недостаточной дозой препарата. Суточная доза дексаметазона в нашем случае не превышала 0,5–0,75 мг/сут. По данным литературы, средние суточные дозы дексаметазона при синдроме резистентности к ГК варьировали от 1 до 3 мг/сут [8]. Кроме того, по данным литературы, даже легкие и субклинические варианты течения заболевания требовали больших доз дексаметазона для достижения компенсации, чем примененные у пациентки [2][8][9][20]. Мы не стали повышать дозу дексаметазона и вовсе отменили этот препарат, так как заподозрили возникновение побочных эффектов терапии (появилась округлость щек, которую расценили как матронизм).

Поскольку пациентку беспокоили проявления гиперандрогении, а электролитных расстройств и артериальной гипертонии у нее не было, пробно назначили терапию антагонистами андрогеновых рецепторов — флутамидом. На этом фоне нормализовался менструальный цикл и уменьшились проявления угревой сыпи. За два года наблюдения на фоне терапии флутамидом сохранялся хороший терапевтический эффект, осложнений терапии не отмечалось. Тем не менее рассматривается возвращение в будущем к терапии дексаметазоном. Целью такой терапии является не только нивелирование клинических проявлений, но и снижение уровня АКТГ. Известны случаи развития аденомы гипофиза у пациентов с тяжелым течением синдрома резистентности к ГК на фоне хронической гиперстимуляции секреции АКТГ [10]. Однако развитие аденом гипофиза описано у больных с тяжелой резистентностью к АКТГ, а у данной пациентки отмечаются высоконормальные или слегка повышенные уровни АКТГ, в связи с чем в настоящее время выбрана наблюдательная тактика в сочетании с терапией флутамидом.

Отказаться от дексаметазона как метода лечения в нашем случае заставили также сомнения в диагнозе — отрицательный результат молекулярно-генетического исследования, сочетание с неясным периодическим тиреотоксикозом без клинических проявлений.

В литературе есть описание пяти случаев синдрома резистентности к глюкокортикоидам без мутаций в гене NR3C1 [13]. У четырех пациентов исследовали функциональную активность рГК в моноцитарных лейкоцитах, и во всех случаях она была снижена. Отмечалось сниженное количество рецепторов, нарушенные аффинность и способность дексаметазона ингибировать митогениндуцированную клеточную пролиферацию. Мы также провели подобное исследование у нашей пациентки. В лаборатории нуклеиновых кислот Института молекулярной биологии РАН оценили показатели функциональной активности рГК в лейкоцитах периферической крови. Нормальные показатели связывания рГК с лигандом, нормальная концентрация рГК, отсутствие нарушений в соотношении α- и β-изоформ рГК исключили патологию собственно рГК как причины синдрома резистентности. В то же время были получены данные о нарушении внутриклеточной передачи сигнала от глюкокортикоидов на этапе переноса комплекса ГК-рГК из цитозоля в ядро клетки-мишени.

Сделать предположения о возможных причинах заболевания в отсутствие патологии рГК позволяют результаты исследований по оценке значимости белков-шаперонов в развитии синдрома резистентности к ГК. Эти исследования обычно проводятся у пациентов с аутоиммунными заболеваниями, нечувствительными к терапии ГК [11][12]. На этом уровне сигнальной трансдукции функционально значимыми являются молекулы, участвующие в трансмембранном переносе рецепторов и защищающие их от ферментативной деградации. В случае с рГК роль молекулярных шаперонов выполняют иммунофиллины (FK506-Binding Immunophillin) FKBP51 и FKBP52 — нарушения в их соотношении приводят к развитию либо ГК резистентности, либо гиперчувствительности [1]. Кроме иммунофиллинов, причиной синдрома резистентности к ГК может быть патология других внутриклеточных белков-шаперонов — белков теплового шока (heat shock protein — HSP90 и HSP70) [15]. Тем не менее возможно, что и в нашем клиническом случае изменение комплексообразования с перечисленными белками-партнерами стало причиной заболевания.

Данные клинических исследований свидетельствуют о супрессивном действии ГК на тиреоидную функцию путем как транс-, так и парагипофизарного взаимодействия [17][18]. Это противоречит длительно наблюдаемому транзиторному тиреотоксикозу у пациентки. Возможно предположить связь нестабильного субклинического тиреотоксикоза с основным заболеванием за счет нарушения механизмов регуляции функции щитовидной железы глюкокортикоидами. Патология белков-шаперонов, играющих роль в передаче сигнала от ТТГ к тиреоцитам [16], также могла бы объяснить связь резистентности к ГК с необычным функциональным нарушением щитовидной железы в виде гипертироксинемии при отсутствии проявлений тиреотоксикоза, однако для подтверждения этой гипотезы необходимы дальнейшие исследования.

## ЗАКЛЮЧЕНИЕ

Синдром первичной резистентности к глюкокортикоидам — это крайне редкое заболевание, характеризующееся нарушением внутриклеточной передачи сигнала от глюкокортикоидов к клеткам-мишеням на уровне рГК или в другом звене передачи сигнала с рецептора. Компенсаторная активация гипоталамо-гипофизарно-надпочечниковой оси приводит к гиперандрогении и гиперсекреции кортикостероидов с минералокортикоидной активностью. Вариабельность тяжести и неспецифичность проявлений синдрома резистентности к ГК может затруднить своевременную диагностику заболевания. При соответствии клинических и лабораторных данных обследования синдрому резистентности к ГК рекомендуется проводить исследование гена рГК NR3C1 как ведущей причины развития синдрома. Отсутствие мутаций в NR3C1 не исключает диагноза, доказать нарушение чувствительности к ГК в этом случае возможно с помощью функционального исследования рГК.

## ДОПОЛНИТЕЛЬНАЯ ИНФОРМАЦИЯ

Источники финансирования. Работа выполнена в рамках государственного задания Минздрава России «Разработка новых технологий диагностики и мониторинга опухолей коры надпочечников с использованием метаболомных и протеомных технологий», рег. No 123021300098-7.

Согласие пациента. Пациентка добровольно подписала информированное согласие на публикацию персональной медицинской информации в обезличенной форме в журнале «Проблемы эндокринологии».

Конфликт интересов. Авторы декларируют отсутствие явных и потенциальных конфликтов интересов, связанных с публикацией настоящей статьи.

Участие авторов. Все авторы одобрили финальную версию статьи перед публикацией, выразили согласие нести ответственность за все аспекты работы, подразумевающую надлежащее изучение и решение вопросов, связанных с точностью или добросовестностью любой части работы.

Благодарности. Выражаем благодарность Орловой Елизавете Михайловне и Каревой Марии Андреевне за помощь в подготовке научной публикации.
